# Repurposing Antipsychotic Agents in Oncology: Mechanistic Rationale, Emerging Evidence, and Therapeutic Potential

**DOI:** 10.3390/cancers18182992

**Published:** 2026-09-16

**Authors:** Adina Qu, Kayla Gray, Sahar Fattani, Sumaiya Ansari, Haneen Hamed, Medhat S. El-Halawany, Debasis Mondal, Maysoon M. Makhlouf, Zakaria Y. Abd Elmageed

**Affiliations:** 1 Department of Biomedical Science, Discipline of Pharmacology, Edward Via College of Osteopathic Medicine (VCOM), 4408 Bon Aire Drive, Monroe, LA 71203, USA; aqu@ulm.vcom.edu (A.Q.); kgray01@ulm.vcom.edu (K.G.); sfattani@ulm.vcom.edu (S.F.); sansari@ulm.vcom.edu (S.A.); hhamed@ulm.vcom.edu (H.H.); mmakhlouf@ulm.vcom.edu (M.M.M.); 2 Division of Natural & Computational Sciences, Texas College, Tyler, TX 75702, USA; melhalawany@txstate.edu; 3 Department of Molecular Sciences, DeBusk College of Osteopathic Medicine, Lincoln Memorial University, Knoxville, TN 37932, USA; debasis.mondal@lmunet.edu

**Keywords:** antipsychotics, drug repurpose, antineoplastic, targeting pathways, clinical applications

## Abstract

Antipsychotic agents—spanning first-, second-, and third-generation drug classes—are emerging as mechanistically diverse candidates for repurposing in oncology. Although developed to modulate dopaminergic and serotonergic neurotransmission, some of these compounds exert pleiotropic effects on cancer cell survival, metabolism, and stemness. Across multiple malignancies, antipsychotics disrupt oncogenic signaling networks including PI3K/Akt/mTOR, STAT3/5, MAPK/ERK, and Wnt/β-Catenin, impair lysosomal and autophagic flux, and induce mitochondrial and calcium-dependent metabolic stress. Notably, several phenothiazines and dopamine-modulating agents selectively reduce cancer stem cell populations, suggesting potential utility in limiting recurrence and mitigating therapeutic resistance. These effects appear to involve disruption of CSC signaling pathways and interference with dopamine-dependent mechanisms that support stemness and survival. Preclinical studies consistently demonstrate antiproliferative, proapoptotic, antimetastatic, and chemosensitizing effects, whereas early clinical investigations demonstrate target engagement and feasibility but do not establish anticancer efficacy. Clinical trials of thioridazine in acute myeloid leukemia and chlorpromazine in glioblastoma show evidence of pharmacodynamic activity in patients, though these findings remain preliminary and non-efficacy-based. However, translation remains constrained by dose-limiting toxicities, pharmacokinetic barriers, and conflicting epidemiologic signals regarding long-term carcinogenic risk. Here, we synthesize mechanistic, translational, and emerging clinical findings relevant to antipsychotic repurposing in oncology, highlight tumor contexts in which these agents may hold potential, and outline future directions including biomarker-guided patient selection, rational combination strategies, and artificial intelligence (AI)-enabled drug-repurposing pipelines. Collectively, antipsychotics represent a promising yet underexplored class of multi-targeted agents; to define their full therapeutic potential will require rigorous mechanistic validation and carefully designed clinical trials.

## Simple Summary

This review summarizes the emerging anticancer activities of antipsychotic agents driven by diverse, non-neurologic mechanisms. These compounds disrupt key signaling pathways, including PI3K/Akt/mTOR, STAT3/f, MAPK/ERK, and Wnt–β-Catenin, and induce lysosomal dysfunction, impaired autophagy, and mitochondrial metabolic stress. Preclinical studies consistently demonstrate antiproliferative, proapoptotic, antimetastatic, and chemosensitizing effects. Early clinical investigations, including thioridazine in leukemia and chlorpromazine in glioblastoma, show preliminary biological activity, although translation remains limited by toxicity, pharmacokinetic barriers, and mixed epidemiologic findings. Future efforts should emphasize biomarker-guided patient selection, rational combination strategies, and AI-enabled drug repurposing approaches to better define the therapeutic potential of these agents.

## 1. Introduction

Drug repurposing has become an increasingly attractive strategy in oncology, offering accelerated development timelines, reduced costs, and established safety profiles [1]. Among repurposing candidates, antipsychotic agents have gained unexpected attention as potential modulators of tumor biology [2]. Although designed to modulate dopaminergic and serotonergic neurotransmission, antipsychotics influence multiple cancer-relevant cellular processes, including proliferation, apoptosis, angiogenesis, metabolic adaptation, and cancer stem cell (CSC) maintenance [3,4,5]. Although several prior reviews have summarized antipsychotic repurposing in oncology [6,7], this review integrates mechanistic, pharmacologic, and translational evidence; emphasizes CSC-associated effects and autophagy disruption; and critically evaluates exposure–response limitations and biomarker-guided strategies.

Initial epidemiologic observations suggested a potential link between antipsychotic exposure and altered cancer risk or progression in psychiatric populations [8]. Conversely, some studies have reported increased gynecologic cancer risk with certain antipsychotics [9], highlighting the need for careful mechanistic and epidemiologic evaluation. Mechanistic studies subsequently revealed that many antipsychotics dysregulate pathways central to tumor survival, including dopamine receptor signaling, phosphoinositide-3-kinase (PI3K)/protein kinase B (Akt)/mammalian target of rapamycin (mTOR), mitogen-activated protein kinase (MAPK)/extracellular signal-regulated kinase (ERK), wingless and Int-1 (Wnt)/β-catenin, and autophagy regulation [10,11,12,13,14]. Accumulating preclinical evidence has sparked growing interest in repurposing selected antipsychotic agents as adjunctive or investigational anticancer therapies, reflecting their emerging mechanistic relevance beyond psychiatric indications. This review synthesizes the pharmacologic classes of antipsychotics, their mechanistic relevance to cancer, preclinical evidence across tumor types, emerging clinical data, and key challenges that must be addressed to advance these agents toward clinical translation.

## 2. Search Strategy and Evidence Selection

This narrative review was based on structured searches of PubMed/MEDLINE, Scopus, and Web of Science conducted in June 2026. Search terms included “antipsychotics,” “phenothiazines,” “anticancer,” “cancer stem cells,” “autophagy,” “drug repurposing,” “preclinical,” “in vitro,” “in vivo,” and “adverse effects.” ClinicalTrials.gov was also queried for ongoing or completed trials. Studies were included if they met criteria relevant to the scope of this review: primary mechanistic studies, in vitro/in vivo oncology models, epidemiologic analyses, and clinical trials evaluating antipsychotics in cancer contexts. The following exclusion criteria were applied: non-oncology psychiatric studies, non-English publications, and studies lacking mechanistic or translational relevance. Evidence was categorized by model type (in vitro, in vivo, observational, phase I/II).

## 3. Classification of Antipsychotic Agents

Antipsychotics are traditionally classified into three major groups based on receptor pharmacology and clinical profile: first-generation (or typical antipsychotics), which primarily act as dopamine D2 receptor (D2R) antagonists; second-generation (or atypical antipsychotics), which generally combine D2R antagonism with 5-hydroxytryptamine receptor 2A (5-HT2A) antagonism; and third-generation antipsychotics, which are characterized mainly by dopamine receptor partial agonist activity. Table 1 summarizes representative agents, indications, adverse effects, and oncology-relevant notes.

## 4. Signaling Pathways Associated with Antipsychotic Effects in Cancer

**Figure 1:**
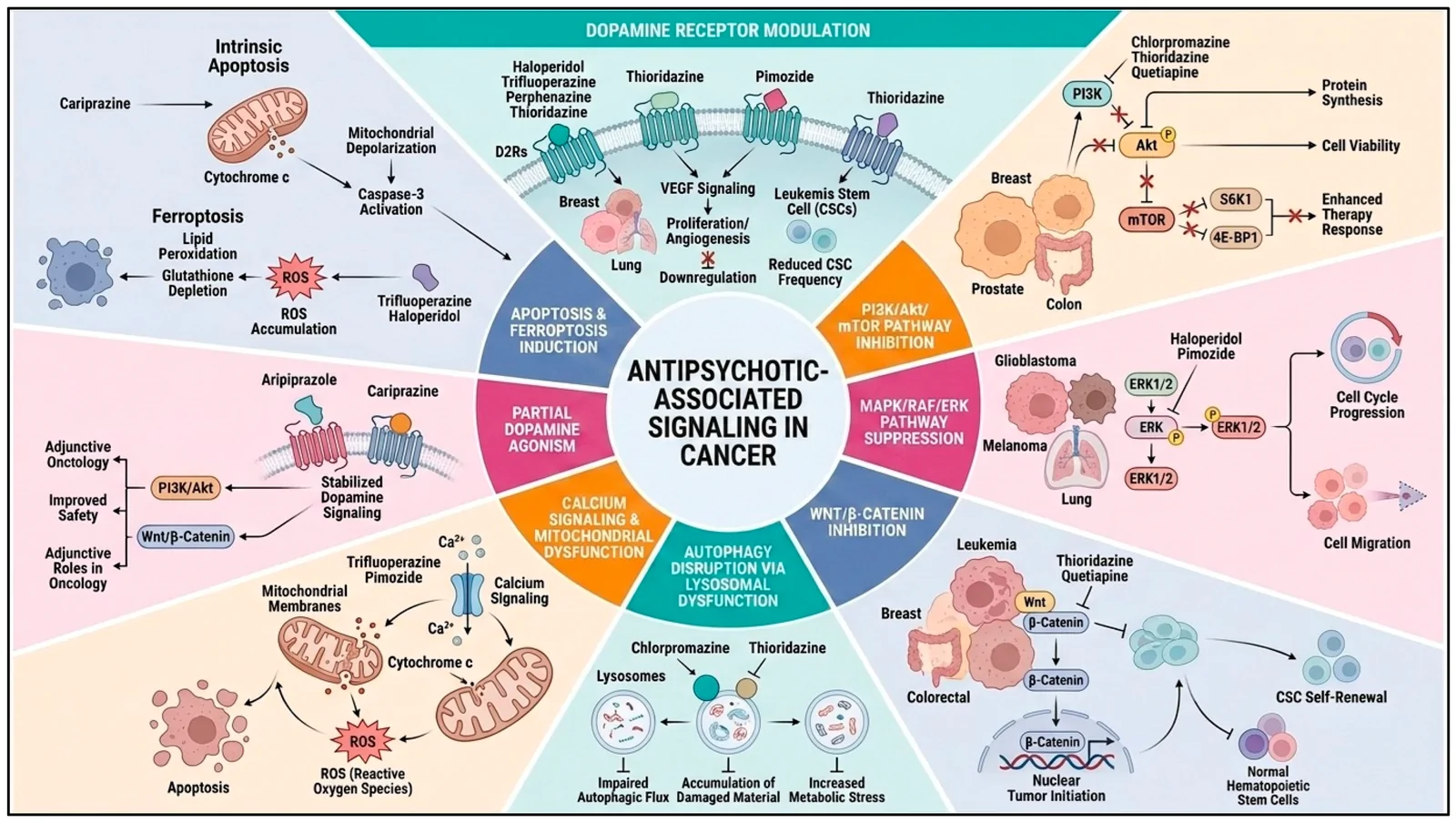
Integrated Signaling Pathway Model of Antipsychotic-Mediated Anticancer Activity. This diagram summarizes antipsychotic-mediated pathways through which these agents exert anticancer effects. Dopamine D2 receptor (D2R) antagonism reduces proliferative and angiogenic signaling and has shown CSC-associated effects in specific preclinical models. The effect of antipsychotics on survival pathways includes PI3K/Akt/mTOR suppression, while MAPK/RAF/ERK inhibition reduces cell cycle progression and migration. Disruption of Wnt/β-Catenin signaling impairs CSC self-renewal and tumor initiation. Lysosomal alkalinization blocks autophagic flux, leading to metabolic stress, whereas calcium dysregulation and mitochondrial destabilization promote reactive oxygen species (ROS) accumulation and intrinsic apoptosis. These mechanisms converge to reduce tumor growth, enhance chemosensitivity, and promote cancer cell death across multiple malignancies. This diagram was generated by FigureLabs (https://figurelabs.ai/ accessed on 3 September 2026), an AI-assisted scientific illustration platform.

Antipsychotic agents exert their anticancer effects through a diverse set of molecular mechanisms that converge on pathways governing proliferation, survival, metabolism, angiogenesis, and CSC maintenance (summarized in Figure 1). These mechanisms have been documented across multiple malignancies, including glioblastoma, leukemia, breast, colorectal, and prostate cancers—and are supported primarily by preclinical evidence. Collectively, they highlight the polypharmacology of antipsychotics and their potential utility as adjunctive or repurposed anticancer agents [7,42]. Below, the antitumor effects of these agents are further highlighted.

### 4.1. Dopamine Receptor Modulation

D2Rs are differentially overexpressed in several cancers, including breast cancer, glioblastoma, lung cancer, and leukemia. Many first-generation antipsychotics—such as haloperidol, trifluoperazine, perphenazine, thioridazine, pimozide, and the second-generation agent risperidone—suppress D2R-mediated signaling. In the context of preclinical studies, this inhibition has been associated with reduced tumor proliferation and angiogenesis, partly through downregulation of vascular endothelial growth factor (VEGF)-dependent pathways [43,44,45]. Thioridazine demonstrates particularly notable selectivity for leukemia stem cells (LSCs), inducing differentiation and reducing CSC frequency [24,46]. Consistent with these findings, D2R blockade appears to contribute to decreased tumor growth and impaired CSC survival in multiple preclinical models [24]. Phenothiazine derivatives such as perphenazine and prochlorperazine suppress glioblastoma cell migration and invasion by modulating multidrug resistance proteins, integrin signaling, and α-tubulin dynamics [47]. These observations support continued investigation of dopamine-modulating antipsychotics in tumors characterized by elevated D2R expression [48].

### 4.2. The PI3K/Akt/mTOR Pathway Dysregulation

The PI3K/Akt/mTOR signaling axis, frequently dysregulated in breast, prostate, and colon cancers, is a central regulator of tumor survival, metabolic reprogramming, and proliferation. Several repurposed antipsychotics, including chlorpromazine, thioridazine, and quetiapine, have been shown to inhibit Akt phosphorylation and its downstream mTOR activity as a consequence of receptor-mediated signaling perturbation, thereby impairing protein synthesis and contributing to suppressed tumor growth [49]. Experimental studies in glioma and breast cancer models consistently demonstrate reduced cancer cell viability, induction of apoptosis, and enhanced sensitivity to standard therapies following pharmacologic inhibition of this pathway [7]. Because the PI3K/Akt/mTOR signaling pathway contributes to aggressive tumor behavior and chemoresistance, these agents may warrant exploration in combination therapy approaches [50].

### 4.3. Modulation of MAPK/RAF/ERK Pathway

The MAPK/rapidly accelerated fibrosarcoma (RAF)/ERK pathway regulates cell cycle progression and proliferation and is frequently activated in glioblastoma, melanoma, and lung cancer. Antipsychotics such as haloperidol and pimozide have demonstrated antiproliferative effects associated with suppression of the ERK1/2 pathway and reduced tumor cell migration [11,16]. In glioblastoma, reduced ERK phosphorylation has been associated with decreased proliferation and shifts in transcriptional programs linked to aggressive tumor behavior, suggesting that these agents may influence oncogenic signaling in an adjunctive, exploratory context [51].

### 4.4. Wnt/β-Catenin-Mediated Pathway

The Wnt/β-Catenin pathway plays a key role in CSC maintenance, metastasis, and treatment resistance in leukemia, breast, and colorectal cancers. Agents such as thioridazine and quetiapine have been associated with suppressed β-Catenin signaling, impaired CSC self-renewal, and reduced tumor initiation capacity [52]. In leukemia models, thioridazine has been reported to selectively affect CSC populations while sparing normal hematopoietic stem cells, a property that may hold relevance for limiting relapse and resistance. Accordingly, modulation of Wnt/β-Catenin signaling may contribute to improving long-term treatment durability in exploratory settings [46].

### 4.5. Autophagy Disruption Through Lysosomal Dysfunction

Autophagy enables cancer cells to survive metabolic stress through degradation and recycling of intracellular components. Several antipsychotics, including chlorpromazine and thioridazine, disrupt lysosomal function and impair autophagic flux, resulting in accumulation of damaged cellular material and increased metabolic stress [18,53,54,55]. Experimental studies have shown that inhibition of protective autophagy sensitizes glioblastoma cells to chemotherapy and promotes apoptosis [53]. This mechanism is particularly relevant in tumors that rely heavily on autophagy for survival under hypoxic or nutrient-limited conditions.

### 4.6. Calcium Signaling and Mitochondrial Dysfunction

Calcium homeostasis is essential for mitochondrial integrity, ATP production, and apoptosis regulation. Antipsychotics such as trifluoperazine and pimozide disrupt intracellular calcium signaling, destabilizing mitochondrial membranes and increasing reactive oxygen species (ROS) in glioblastoma and leukemia cells [7,56]. These effects promote cytochrome c release and activation of intrinsic apoptotic pathways. Rapidly proliferating tumor cells, which exhibit heightened metabolic demands, appear particularly vulnerable to this form of mitochondrial stress [57].

### 4.7. Partial Dopamine Agonism

Third-generation antipsychotics, including aripiprazole and cariprazine, function as partial dopamine agonists, stabilizing dopamine signaling rather than fully blocking receptor activity. In cancers with dysregulated dopamine pathways, this modulation may reduce abnormal proliferative signaling while reducing toxicity associated with complete D2R antagonism [38,39]. These agents also influence PI3K/Akt and Wnt/β-Catenin signaling and may offer improved safety profiles compared with first-generation antipsychotics, supporting their exploration as adjunctive modulators of oncology. In conclusion, antipsychotics across all drug classes converge on multiple cancer-relevant pathways, target and deplete the CSC population, disrupt metabolic resilience, and sensitize tumors to standard therapies, collectively underscoring their potential as repurposed anticancer agents. These mechanistic insights provide a strong foundation for translational investigation and support the development of rational, pathway-directed combination strategies.

### 4.8. Apoptosis and Ferroptosis

Antipsychotics exhibit diverse anticancer mechanisms, prominently including apoptosis and ferroptosis. Several agents activate intrinsic apoptotic pathways through mitochondrial dysfunction, cytochrome c release, and caspase-3 activation. The ability of phenothiazine derivatives to induce necroptosis in normal versus cancer cell lines appears to be highly concentration-dependent and influenced by the tumor-specific context, including cellular phenotype and pathway activation status [58]. For example, cariprazine induces dose-dependent cytotoxicity and robust mitochondrial apoptosis in HeLa cells, characterized by membrane depolarization, increased Bax/Bcl-2 ratio, and caspase-3 activation [40]. In parallel, multiple antipsychotics trigger ferroptosis, an iron-dependent form of cell death driven by lipid peroxidation and impaired glutathione metabolism. Trifluoperazine induces ferroptosis in acute myeloid leukemia (AML) and oral cancer cells by suppressing the nuclear factor erythroid 2-related factor 2 (Nrf2)/solute carrier family 7 member 11 (SLC7A11)/glutathione Peroxidase 4 (GPX4) axis, elevating lipid-derived ROS, and promoting autophagy-linked oxidative injury [59]. Haloperidol similarly activates ferroptotic signaling in breast cancer cells, increasing lipid peroxidation, ROS accumulation, glutathione depletion, and mitochondrial membrane potential loss, consistent with non-canonical ferroptosis pathways [60].

In summary, antipsychotics primarily act through direct binding to neurotransmitter receptors, and the downstream changes observed in ERK, PI3K/AKT/mTOR, STAT3/5, and Wnt/β-catenin pathways reflect indirect signaling consequences rather than direct kinase targeting. This distinction between direct target binding, downstream signaling, and stress-induced changes is essential for accurate mechanistic interpretation.

## 5. Preclinical Evidence

Abundant preclinical evidence highlights the anticancer potential of antipsychotics across a variety of malignancies. Both in vitro and in vivo studies demonstrate that these drugs can modulate key oncogenic pathways, induce apoptosis, autophagy, and cell cycle arrest, inhibit tumor progression, invasion, and metastasis, and enhance the efficacy of standard chemotherapeutic regimens. Their most credible role in oncology is as investigational adjunctive agents, particularly as chemosensitizers, radiosensitizers, CSC modulators, or targeted modulators of tumor-specific molecular pathways. A selected list of these agents is further discussed and summarized in Table 2.

### 5.1. Thioridazine

Evidence for selective anticancer activity varies substantially across antipsychotic agents and cancer models. Among first-generation antipsychotics, thioridazine (TDZ) has demonstrated preferential cytotoxicity toward CSCs while sparing normal hematopoietic stem cells [46]. Mechanistically, TDZ exerts its effect through dopamine receptor antagonism, thereby disrupting CSC-associated signaling pathways that support self-renewal and therapeutic resistance. Subsequent studies have extended these observations across multiple tumor types. In glioblastoma, TDZ inhibits CSC proliferation and induces autophagy by modulating survival pathways, including PI3K/Akt signaling [23]. Earlier studies have also demonstrated that TDZ suppresses proliferation in human breast cancer cell lines, supporting its broader anticancer activity [61]. Furthermore, TDZ exhibits cytotoxic and anti-proliferative effects in ovarian cancer models, reinforcing its potential across multiple tumor types [62]. It also targets autophagy-dependent survival mechanisms in osteosarcoma CSCs, highlighting its role in disrupting tumor maintenance and resistance [4]. Additionally, emerging evidence indicates that TDZ induces apoptosis in gastric cancer cells by regulating B-cell lymphoma 2 (Bcl-2) family proteins and both proliferative and apoptotic markers, further suggesting its multi-tumor applicability [63].

### 5.2. Chlorpromazine

Chlorpromazine (CPZ), a prototypical first-generation phenothiazine antipsychotic, has been extensively investigated for its anticancer properties across multiple cancer types. In glioma cells, CPZ induces apoptosis and autophagy by disrupting mitochondrial membrane potential, increasing ROS production, and suppressing PI3K/Akt/mTOR signaling [10]. Additionally, CPZ has been shown to inhibit clathrin-mediated endocytosis, a process critical for receptor recycling and oncogenic signaling, thereby impairing tumor cell survival [17]. In hepatocellular carcinoma, CPZ induces cell cycle arrest (primarily at the G2/M phase) and promotes oxidative stress-mediated cytotoxicity [17]. Other studies suggest that it interferes with DNA repair mechanisms and may sensitize tumor cells to radiotherapy and chemotherapy [64].

### 5.3. Pimozide

Pimozide (PZ) has also demonstrated significant anticancer activity, particularly by inhibiting oncogenic signaling pathways. Its mechanistic role of blocking D2Rs to treat chronic schizophrenia and other psychotic disorders can be repurposed for use in oncology. Earlier studies identified its roles in suppressing signal transducer and activator of transcription 5 (STAT5) signaling in hematologic and solid malignancies [26]. Notably, PZ has shown promising effects in prostate cancer cells, where inhibition of STAT5 signaling reduces tumor cell proliferation and increases apoptosis, highlighting STAT5 as a critical therapeutic target in prostate cancer [65]. Moreover, a 2024 study showed that PZ promotes apoptosis and enhances autophagy in breast cancer cells by modulating the RAF/ERK1/2 pathway, reinforcing its therapeutic potential in hormone-responsive tumors [11]. Additional evidence suggests that PZ may also inhibit STAT3 signaling and reduce cancer cell migration and invasion [25].

### 5.4. Risperidone and Olanzapine

In addition to first-generation antipsychotics, second-generation antipsychotics such as risperidone (RD) and olanzapine (OP) have also shown modulatory effects on tumor-associated signaling pathways. Second-generation antipsychotics, while primarily developed to reduce extrapyramidal side effects, have also demonstrated anticancer properties. RD has been reported to inhibit proliferation and induce apoptosis in breast cancer cell lines via D2R antagonism and downstream signaling pathways [27]. Some studies also suggest potential effects on angiogenesis and on the regulation of the tumor microenvironment [27].

Although OP exhibits limited direct cytotoxic effects, it may improve chemotherapy tolerability and indirectly enhance therapeutic outcomes, particularly in supportive oncology settings [61]. It is widely used to manage chemotherapy-induced nausea and vomiting (CINV) and may indirectly improve treatment adherence and patient outcomes [66]. Emerging preclinical evidence indicates that OP can modulate survival-related signaling in tumor cells, including reducing survivin expression in lung, pancreatic, and ovarian cancer models and thereby enhancing sensitivity to chemotherapeutic agents [67]. However, despite these pathway-level effects, its direct anticancer activity remains limited and unvalidated in clinical settings.

### 5.5. Aripiprazole and Brexpiprazole

Third-generation antipsychotics, defined by their partial agonism at dopamine D2 receptors, are gaining attention as repurposing candidates in oncology. Aripiprazole (APZ), the most widely studied agent in this class, has demonstrated anticancer activity in several preclinical models. Mechanistic studies summarized in a recent systematic review show that APZ suppresses tumor cell proliferation and induces apoptosis by modulating PI3K/Akt/mTOR and Wnt/β-Catenin signaling, while concurrently triggering mitochondrial dysfunction and endoplasmic reticulum stress [12]. APZ also overcomes chemoresistance by inhibiting P-glycoprotein activity and expression, thereby enhancing the cytotoxic efficacy of chemotherapy and radiotherapy in preclinical studies. Earlier findings further support its role in modulating CSCs and reversing drug resistance, suggesting its potential to prevent tumor recurrence [46].

Similarly, brexpiprazole (BPZ) has demonstrated promising anticancer effects, including suppression of tumor growth and induction of apoptosis in glioblastoma, pancreatic, and lung cancer models. These effects are associated with downregulation of survivin and reduction in CSC markers such as sex determining region Y-box 2 (Sox2), with evidence of selective cytotoxicity toward cancer cells and tumor inhibition in vivo [36]. Furthermore, BPZ has been reported to sensitize glioma stem cells to targeted therapies, including epidermal growth factor receptor (EGFR) inhibitors, further supporting its role in combination treatment strategies. Although less extensively studied, other third-generation agents, such as cariprazine, may exhibit similar anticancer potential through dopaminergic modulation and effects on tumor-related signaling pathways; however, current evidence remains limited, warranting further investigation.

Collectively, these preclinical findings highlight the diverse anticancer mechanisms of antipsychotic drugs, including CSC-associated effects, inhibition of oncogenic signaling pathways such as PI3K/Akt and STAT3/5, induction of apoptosis and autophagy, and modulation of tumor metabolism. Importantly, recent studies continue to reinforce and expand on these mechanisms across multiple cancer types, strengthening the rationale for further translational and clinical investigation of antipsychotics as repurposed anticancer agents. The anticancer potential spans across three generations of antipsychotics, with first-generation antipsychotics generally showing the most robust direct cytotoxic effects in preclinical models. Third-generation antipsychotics have been proposed to offer potential tolerability advantages, but evidence supporting safety or therapeutic advantages in oncology settings remains limited and largely preclinical. However, most studies do not include systematic comparison with normal cell lines or provide therapeutic index data. In many models, the concentrations required to achieve anticancer effects exceed clinically attainable levels, making it difficult to determine true selectivity. Taken together, these findings offer preliminary support for continued translational and clinical investigation of antipsychotics as potential repurposed agents in cancer therapy.

## 6. Clinical Evidence and Trials

While clinical data remains limited (summarized in Table 3), emerging retrospective analyses and early-phase trials suggest that antipsychotic exposure may be associated with reduced cancer incidence, slower disease progression, or improved treatment response in certain patient populations. Population-based and pharmacoepidemiologic studies have reported association between antipsychotic exposure and cancer outcomes, including lower overall cancer incidence among individuals receiving long-term antipsychotic therapy, particularly with phenothiazines and other dopamine-modulating agents, after adjusting for psychiatric comorbidities and lifestyle factors [68,69]. Additional analyses suggest correlation between antipsychotic use and reduced mortality in specific cancers, such as breast and lung cancer, although these findings remains observational and cannot establish causality [69]. Early translational studies demonstrate that certain antipsychotics engage relevant oncologic targets in vivo. For example, thioridazine has demonstrated activity against LSCs and is currently being evaluated in clinical studies for AML and other hematologic malignancies [46,70]. These findings indicate biological activity but do not constitute clinical efficacy. Phase I/II studies provide preliminary evidence of antitumor activity but remain exploratory. Additional trials are investigating phenothiazines and related agents in solid tumors, including glioblastoma and prostate cancer. Collectively, these findings—spanning population-level observations, supportive-care oncology trials, and early mechanistic clinical studies—underscore the growing interest in antipsychotics as adjunctive or repurposed anticancer agents, while highlighting the need for rigorously designed prospective trials to define their true clinical utility.

### 6.1. Clinical Applications of Antipsychotics

#### 6.1.1. Thioridazine

A phase I clinical trial (NCT02096289) in adults with relapsed or refractory acute AML used a standard dose-escalation design to evaluate the safety and biologic activity of TDZ, in combination with cytarabine. The study demonstrated modest anti-leukemic activity, but its use was limited by toxic effects, including QT prolongation and neurological symptoms. While no patients achieved complete remission, results demonstrated leukemia cell differentiation, reduced LSCs, and D2R engagement in human AML cells [70]. These preliminary findings are consistent with preclinical studies showing CSC-related effects mediated through D2R pathways. Together, they suggest that dopamine-receptor-based strategies may warrant further evaluation in biomarker-selected AML populations [46].

#### 6.1.2. Chlorpromazine

CPZ has demonstrated a broad spectrum of preclinical antitumor activity, including reductions in tumor size and weight in animal models, inhibition of cell proliferation, and disruption of key oncogenic signaling pathways—PI3K/Akt/mTOR, MAPK, and Yes-associated protein (YAP)—that regulate tumor growth, survival, and therapeutic resistance [64,73]. The initiation of a formal clinical trial may strengthen the rationale for repositioning CPZ as an adjunctive cancer therapy, providing an opportunity to determine its practical benefit for patients and validate its mechanistic effects in a clinical setting. The RACTAC phase II trial (ClinicalTrials.gov ID NCT04224441) evaluated CPZ at a dose of 50 mg/day, administered concomitantly with temozolomide in glioblastoma patients with hypo-methylated O6-methylguanine-DNA-methyltransferase (MGMT) promoters. Among 41 enrolled patients, 66% remained progression-free at 6 months, reported as the study’s primary endpoint [71]. In 10% of patients, the CPZ dose required reduction due to treatment-associated adverse effects. Limitations included a small sample size, lack of randomization, and absence of pharmacokinetic monitoring.

#### 6.1.3. Olanzapine

OP plays a fundamentally different role in oncology, serving as a supportive care medication for chemotherapy-induced nausea and vomiting (CINV) rather than functioning as an agent that directly targets cancer cells. A Phase III randomized, double-blind, placebo-controlled trial (NCT02116530) involving 401 patients evaluated the addition of OP to standard antiemetic therapy, dexamethasone, a 5-hydroxytryptamine type 3 (5-HT3) receptor antagonist, and a neurokinin-1 (NK1) receptor antagonist, for the prevention of chemotherapy-induced nausea and vomiting. The trial demonstrated a significant improvement in nausea control, with OP-treated patients experiencing fewer adverse effects and meaningful gains in quality of life compared with placebo [28,29]. Sedation emerged as the most prominent adverse effect in OP-treated patients, although it remained generally manageable within the trial context. Despite these encouraging findings, OP has not undergone systematic evaluation at different dosing levels or multiple chemotherapy cycles, leaving its cumulative safety profile undefined.

#### 6.1.4. Aripiprazole

The association between APZ use and subsequent breast cancer risk among individuals with schizophrenia was investigated through a retrospective cohort design [72], which relied on nationwide insurance claims data rather than a registered clinical trial. In contrast to the previously discussed medications, which act as dopamine antagonists, APZ functions as a partial D2 agonist and is associated with lower prolactin levels. Because elevated prolactin can stimulate breast tissue proliferation, the use of a third-generation antipsychotic such as APZ may be linked to a reduced risk of breast cancer [72]. This further supports the idea that antipsychotic medications may have differing effects on cancer risk based on their mechanism of action.

Overall, these studies demonstrate that antipsychotics may influence cancer through multiple pathways, including direct anti-tumor effects, modulation of CSCs, enhancement of chemotherapy efficacy, and hormonal regulation. However, the variability in study design, patient populations, and mechanisms of action necessitates further patient-based research to better define their potential benefit in cancer therapy. Clinical evidence is promising but insufficient, and more trials are needed.

### 6.2. Challenges in Clinical Translation

Despite promising preclinical results, the clinical translation of antipsychotics faces several hurdles. First, most antitumor effects of antipsychotics occur at concentrations exceeding clinically achievable plasma levels, and dose escalation is limited by QT prolongation, extrapyramidal symptoms (EPS), and metabolic toxicity [74]. Second, high protein binding and limited tumor penetration may reduce the effective exposure of these agents at the tumor site [75]. Third, multiple mechanisms of action across antipsychotic classes complicate biomarker selection and patient stratification. Fourth, epidemiologic studies remain confounded by indication, comorbidities, and lifestyle factors [76]. Finally, reproducibility of preclinical findings varies across cancer models, and negative or null results are underreported. These challenges underscore the need for rigorous pharmacokinetic-pharmacodynamic evaluation, biomarker-guided trial design, and careful toxicity monitoring in future studies.

Many antipsychotics exhibit anticancer half-maximal inhibitory concentration (IC_50_) values from 5 to 50 µM, exceeding clinically achievable plasma concentrations, which typically range from 0.1 to 1 µM. High protein binding (>90%), limited tumor penetration, and dose-limiting QT prolongation further restrict therapeutic exposure. Thioridazine’s anticancer IC_50_ value (~10–20 µM) exceeds its tolerable human Cmax (~0.5 µM). CPZ shows similar discrepancies between in vitro potency and achievable systemic exposure [77]. Aripiprazole and brexpiprazole achieve higher CNS penetration but still fall below most reported anticancer IC_50_ values. These exposure–response gaps represent a major barrier to antipsychotic repurposing.

## 7. Current Challenges and Knowledge Gaps

The rationale for evaluating antipsychotics in oncology extends beyond their established use in managing psychiatric comorbidities among cancer patients [78]. An expanding body of preclinical and translational research has revealed a promising association between antipsychotic agents and anticancer activity, although most evidence to date remains at the experimental or early-phase clinical level [79]. Conversely, some epidemiologic studies have raised concerns regarding a potential carcinogenic risk associated with long-term exposure to certain antipsychotics [80]. It is noteworthy that interest in repurposing antipsychotics for their antitumor potential remains strong—particularly in brain tumors, where many psychiatric agents readily cross the blood–brain barrier, providing a pharmacological advantage in a setting limited by poor drug penetration and the modest effectiveness of current standard-of-care treatments [81].

The inconsistent findings regarding these antipsychotic drugs in cancer cells may reflect their concentration-dependent characteristics, as their biological effects can vary substantially with dose. However, increasing the dose regardless of the drug class can have negative effects on cancer cells and patient outcomes, such as severe neurological, cardiac, or metabolic issues (Summarized in Table 4). Although preclinical evidence supports the dual role of antipsychotics in cancer treatment, typical antipsychotics can have significant side effects that may not be optimal for cancer patients with increased morbidity [82]. Observational studies have reported associations between antipsychotic exposure at higher doses and increased breast cancer incidence and mortality [83]. However, these findings do not establish a causal relationship, and some of the observed antipsychotic use associated with higher breast cancer mortality was likely due to confounding by indication [84]. Even though data on the anticancer potential of third-generation antipsychotics are extremely limited, these findings highlight the importance of administering antipsychotics in appropriate doses and carefully titrating to minimize adverse effects. The anticancer effects of antipsychotics may require higher doses than those clinically indicated in psychiatric applications; this may increase the risks of adverse effects including QT prolongation, EPS, metabolic syndrome, sedation, and hepatic injury. TDZ is of particular concern because it produces dose-dependent QTc prolongation, as documented in drug-specific cardiac safety studies [85,86]. Leonard et al. conducted a retrospective cohort analysis of six antipsychotic users aged 30–70 years across six U.S. states and found that haloperidol and CPZ were associated with a higher risk of cardiac death compared with olanzapine [87].

Consequently, identifying biomarkers in patients can help predict their responsiveness to antipsychotics, thereby optimizing the potential anticancer effects (summarized in Table 5). Potential biomarkers may include D2R overexpression, which is reported in glioblastoma, breast cancer, and leukemia, and correlates with sensitivity to phenothiazines and butyrophenones. CSC-associated markers, such as cluster of differentiation 44 (CD44), aldehyde dehydrogenase 1 (ALDH1), and Sox2/Survivin, are reduced following thioridazine and brexpiprazole treatment in preclinical models [36,46]. Autophagy-related signatures, including microtubule-associated protein 1 light chain 3B (LC3B) accumulation and impaired lysosomal acidification, may predict sensitivity to chlorpromazine and thioridazine. STAT3 activation may identify tumors responsive to pimozide in triple-negative breast cancer and brain tumors [95]. Such biomarkers can help identify the patients who are most likely to benefit while minimizing unnecessary exposure and side effects. Although preclinical data display potential anticancer activity, there are no confirmed biomarkers used clinically for antipsychotic therapy. Therefore, the off-label use of these drugs in oncology requires careful oversight, patient-informed consent, monitoring, and a risk-benefit assessment.

Drug interactions between antipsychotics and chemotherapy are clinically important because many agents share overlapping toxicities and metabolic pathways. Antipsychotics such as clozapine can exacerbate chemotherapy-induced myelosuppression, creating additive risk for neutropenia, which is well documented when clozapine is combined with cytotoxic regimens like cyclophosphamide, doxorubicin, vincristine, and related agents [88,90]. Chemotherapeutic drugs that inhibit the cytochrome P450 enzymes CYP2D6 and CYP3A4 can also increase serum concentrations of antipsychotics, raising the risk of QT prolongation, sedation, or neurotoxicity. Conversely, some psychotropics inhibit CYP enzymes and can elevate levels of anticancer drugs, requiring careful dose adjustments [99]. Additionally, certain combinations—such as phenothiazine antipsychotics with tamoxifen—can increase QT-interval prolongation risk, while agents like haloperidol may show enhanced photosensitivity when paired with methotrexate [99]. Despite these risks, continuation of antipsychotics, including clozapine, is often feasible with close monitoring and supportive measures such as granulocyte colony-stimulating factor (G-CSF), helping maintain psychiatric stability without compromising cancer treatment adherence [89].

Regulatory and economic barriers significantly limit antipsychotic repurposing for cancer therapy, despite growing preclinical evidence. Because new oncology indications require full U.S. Food and Drug Administration (FDA) regulatory pathways—including dose-finding studies, toxicology reassessment, and Phase I–III clinical trials—generic antipsychotics face the same development burden as novel agents but without the protection of market exclusivity [100]. This creates a well-recognized “no profit, no development” problem in drug repurposing, where pharmaceutical companies lack incentive to invest substantial resources in trials for drugs they cannot patent or meaningfully monetize [101]. Additional barriers include limited federal funding streams dedicated to repurposing antipsychotic medications, concerns about cardiac, metabolic, and hematologic toxicities in cancer patients, and fragmented regulatory frameworks that do not streamline approval for new indications of established drugs [102]. As a result, progress in this area relies heavily on academically driven trials, philanthropic support, and public–private partnerships rather than traditional industry pipelines.

Associations between antipsychotic exposure and cancer outcomes must be interpreted with caution because they are heavily influenced by underlying psychiatric comorbidities, lifestyle factors, and treatment-adherence patterns that differ markedly from the general population [103]. Individuals with schizophrenia and other severe mental illnesses exhibit disproportionately high rates of tobacco use, obesity, diabetes, poor diet, and physical inactivity, all of which independently worsen cancer incidence, stage at diagnosis, and survival [104]. Psychiatric symptoms—including cognitive impairment, negative symptoms, and disorganized thinking—contribute to delayed help-seeking, reduced participation in screening programs, and difficulties navigating complex oncology care pathways [105]. These same factors also impair medication adherence, affecting both antipsychotic continuity and adherence to chemotherapy, radiation, or targeted therapies [106]. Moreover, antipsychotic use often correlates with greater psychiatric illness severity, introducing substantial confounding by indication that persists even after statistical adjustment [107]. Hwang et al. (2024) reported that antipsychotic use in the Republic of Korea was generally linked to higher mortality, although patients with psychiatric histories and long-term exposure fared better during radiotherapy, whereas initiating these agents during treatment corresponded to worse outcomes, particularly in breast cancer, where the clinical factors prompting antipsychotic use may themselves contribute to the elevated mortality risk [108]. Together, these behavioral, clinical, and structural determinants underscore the need for prospective, mechanistic, and carefully controlled studies to determine whether antipsychotics exert true biological effects on tumor behavior or whether observed epidemiologic associations primarily reflect the broader health complications experienced by individuals with severe mental illness.

## 8. Perspective on Future Directions

To advance antipsychotics toward clinical translation and their repositioning toward safe and effective cancer therapy, future work should prioritize the following:

### 8.1. Mechanistic Understanding and Perturbational Response

Future directions in repurposing antipsychotics for cancer therapy require deeper mechanistic investigation to define the molecular pathways underlying their antitumor activity. Although several antipsychotics modulate key oncogenic signaling networks—including PI3K/Akt/mTOR, Wnt/β-catenin, and MAPK/ERK—the precise nodes of pathway engagement and their context-specific relevance remain incompletely understood [2,109]. Elucidating whether these effects arise through D2R inhibition, cAMP modulation, downstream G-protein signaling, or through off-target interactions such as mitochondrial disruption, lysosomal stress, or inhibition of kinases and phosphatases will be essential for improving therapeutic precision [110,111]. Parallel efforts should focus on dissecting how antipsychotics influence CSC biology, given emerging evidence that these agents suppress CSC markers, impair self-renewal pathways, and sensitize tumors to standard therapies [112]. Although several antipsychotics have demonstrated CSC-directed effects in preclinical studies, these findings are not uniform across the drug class and vary substantially by agent, tumor type, and experimental model. Such mechanistic clarity is critical for advancing antipsychotics from empirical repurposing candidates to mechanistically validated, precision-guided anticancer therapeutics.

### 8.2. Combination Therapy Strategies

Combination therapy strategies that integrate repurposed antipsychotics with targeted agents or radiotherapy are supported by convergent mechanistic evidence demonstrating synergy across metabolic, signaling, and DNA-damage pathways. Several antipsychotics—including phenothiazines, butyrophenones, and serotonin inverse agonists—modulate PI3K/Akt/mTOR, MAPK, mitochondrial respiration, and oxidative-stress responses, creating a cellular environment that increases susceptibility to targeted inhibition [112,113]. Preclinical studies show that antipsychotics elevate ROS, disrupt autophagy, and impair mitochondrial function, thereby lowering the apoptotic threshold and enhancing the cytotoxicity of PI3K inhibitors in resistant tumors [114]. Parallel evidence indicates that antipsychotics potentiate radiotherapy by increasing oxidative stress, inhibiting DNA-damage repair proteins such as ataxia-telangiectasia mutated and Rad3-related (ATM/ATR), and altering chromatin accessibility, which amplifies radiation-induced double-strand breaks [115]. Together, these mechanisms provide a strong rationale for combining antipsychotics with PI3K inhibitors or radiotherapy to overcome therapeutic resistance, exploit synthetic vulnerabilities, and achieve deeper and more durable antitumor responses.

Clinical considerations—including withdrawal, relapse risk, and long-term tolerability—also influence the translational use of antipsychotics in oncology. A hyperbolic tapering strategy, with dose reductions, has been proposed to limit withdrawal-related adverse effects [116]. This approach encourages individualized decisions about prolonged antipsychotic use by balancing therapeutic benefit against cumulative risk. These principles are relevant when repurposing antipsychotics in cancer therapy, reinforcing the need for dosing strategies that balance mechanistic synergy with patient safety and treatment sustainability. Because antipsychotics engage dopamine, serotonin, and other receptors in extraneural tissues, systemic administration can produce substantial metabolic and cardiovascular adverse effects. Targeted delivery strategies may enhance tumor-specific drug exposure while reducing off-target receptor engagement in healthy tissues [117].

### 8.3. AI-Driven Drug Repurposing and Computational Screening of Antipsychotic Agents for Cancer

Artificial Intelligence (AI)-driven drug repurposing has emerged as a powerful strategy for accelerating the discovery of anticancer therapeutics, particularly among neuropsychiatric and antipsychotic agents whose polypharmacology lends itself to oncologic repositioning. Machine-learning frameworks integrate chemogenomic, transcriptomic, and network-based inference models to systematically map drug–target–disease relationships and uncover previously unrecognized anticancer mechanisms within FDA-approved antipsychotics [118]. These approaches incorporate predictive modeling of drug–protein interactions, large-scale perturbation signatures, and virtual docking against cancer-relevant signaling pathways to prioritize compounds capable of modulating apoptosis, cell-cycle regulation, ferroptosis, and metabolic stress responses [119]. Multi-omics similarity profiling and AI-enabled phenotypic screening further refine the identification of antipsychotic scaffolds that mimic or reverse oncogenic transcriptional programs [120].

Growing international initiatives are expanding AI-enabled drug repurposing in oncology by deploying advanced machine-learning systems that integrate genetic, multi-omics, and pharmacologic datasets with extensive drug libraries and predicted molecular targets to identify repurposed FDA-approved agents and pipeline candidates [121]. The nonprofit Every Cure is advancing AI-driven repurposing candidates through its MATRIX platform, which systematically prioritizes high-value therapeutic candidates and supports early-stage evidence generation; however, its role remains focused on computational nomination and strategic triage rather than established clinical validation of antipsychotic oncology applications. The NIH’s All of Us Research Program strengthens these efforts by providing the world’s largest integrated genomic–clinical dataset, addressing key data limitations that previously hindered oncology repurposing. In Europe, organizations such as Reboot Rx apply AI-powered evidence synthesis to identify and prioritize low-cost generic drugs with anticancer potential, supported through partnerships with national cancer institutes and health systems. Collectively, these initiatives underscore how AI-driven computational screening provides a scalable, cost-efficient framework for uncovering the anticancer potential of antipsychotic agents and advancing their translation into mechanistically informed therapeutic candidates for aggressive cancers.

### 8.4. Personalized Medicine

Personalized medicine approaches integrating pharmacogenomics and tumor profiling offer a rational framework to optimize the use of repurposed antipsychotics in oncology. Pharmacogenomic characterization of drug-metabolizing enzymes, transporters, and receptors has already transformed precision dosing and treatment selection in multiple therapeutic areas, providing a template for biomarker-driven deployment of antipsychotic agents in cancer [122]. In this context, future research should prioritize identification and validation of predictive biomarkers—such as dopamine receptor expression patterns, downstream dopamine signaling signatures, and CSC markers—that may stratify patients according to likelihood of response to dopaminergic modulation [5]. Integration of pharmacogenomic data with multi-omics tumor profiling and microenvironmental features could enable construction of composite biomarker panels that guide selection, dosing, and combination partners for antipsychotic-based regimens while minimizing toxicity and off-target neuropsychiatric effects. Such an approach aligns with broader precision oncology paradigms, in which molecularly defined subgroups receive tailored therapies informed by robust predictive biomarkers rather than empiric, one-size-fits-all treatment algorithms.

### 8.5. Expanded Clinical Trials

Expanded clinical evaluation of repurposed antipsychotics in oncology will require rigorously designed, multi-center trials across diverse cancer types, molecular subgroups, and disease stages to move beyond the current predominance of in vitro and early-phase data. Large-scale phase II–III studies are needed to define the true antitumor efficacy, safety profile, and long-term outcomes of typical and atypical antipsychotics when used as monotherapy or in combination with standard chemo-, radio-, or targeted therapies [73]. Such trials should incorporate stratification by tumor histology, prior treatment exposure, and relevant comorbidities, while prospectively capturing cardiometabolic and neuropsychiatric adverse events that may be accentuated in oncology populations. Cardiometabolic side effects are more pronounced with second-generation antipsychotics; however, despite being a class-wide concern, the degree of risk varies substantially among individual agents [123]. Future studies must also establish standardized dosing regimens, schedules, and combination schemas, informed by pharmacokinetic–pharmacodynamic modeling, and embed patient-reported outcome measures to evaluate symptom burden, functional status, and quality of life in patients receiving these agents. This evidence base is essential to transition antipsychotics from promising repurposing candidates to clinically integrated components of evidence-based cancer care.

## 9. Conclusions

Several antipsychotics demonstrate reproducible preclinical anticancer activity across diverse experimental models. The tested agents exhibited in vitro antitumor activity by modulating dopamine receptor signaling, inhibiting key oncogenic pathways, disrupting autophagy and mitochondrial function, and selectively targeting cancer stem cells. While preclinical evidence is compelling, clinical antitumor efficacy remains unproven, limited by exposure constraints, toxicity considerations, and sparse prospective trials. Carefully designed biomarker-guided studies are required to determine whether these agents can be safely and effectively repurposed. With appropriate supporting data, these agents might be assessed in a complementary or adjunctive capacity alongside current standard therapies.

## Figures and Tables

**Table 1:** Representative Antipsychotic Agents: Clinical Findings, Adverse Effects, and Oncology-Relevant Notes.

Class	Drug	Indications	Adverse Reactions	Relevance to Oncology	References
Typical (1st Generation)	Haloperidol	Psychosis, Tourette syndrome	QT prolongation, Tardive dyskinesia (TD)	Induces apoptosis and inhibits migration in glioblastoma cells. This effect increased with temozolomide and radiation.	[15,16]
	Chlorpromazine	Schizophrenia, intractable hiccups, nausea	Anticholinergic effects, Jaundice, weight gain	Inhibits PI3K/Akt/mTOR and disrupts autophagy in glioma cells, and reduces tumor volume in xenograft model.	[10,17,18]
	Trifluoperazine	Psychosis, schizophrenia	Neuroleptic malignant syndrome (NMS), tardive dyskinesia	Reduces prostate cancer and fibrosarcoma cell invasion through suppression of phosphorylated Akt and β-catenin with minimal effects on murine fibroblasts.	[19,20]
	Perphenazine	Acute psychosis, nausea, and vomiting	Orthostatic hypotension, neuroleptic malignant syndrome	Induces cell cycle arrest and apoptosis in gastric cancer cells and antitumor activity in mouse model. Inhibits cell proliferation and induces apoptosis in endometrial cancer cells and reduces xenograft tumor growth	[21,22]
	Thioridazine	Psychosis	QT prolongation, tardive dyskinesia	Induces autophagy in glioblastoma cells/CSCs and upregulates AMPK activity. Suppresses glioblastoma in vivo. Selectively reduces growth of lung cancer stem-like cells in vitro and in vivo	[23,24]
	Pimozide	Tourette syndrome, schizophrenia	QT prolongation	Inhibits STAT5, STAT3, RAF/ERK pathways, and induces ROS production in breast cancer, CML, and osteosarcoma cells	[11,25,26]
Atypical (2nd Generation)	Risperidone	Bipolar disorder, autism-related irritability	Cerebrovascular events, weight gain	Inhibits triple-negative breast cancer cells in silico, in vitro, and in ovo CAM model through inhibition of VEGFR2	[27]
	Olanzapine	Schizophrenia, bipolar disorder	Hyperglycemia, suicidal ideation	Supportive care in chemotherapy-induced nausea and vomiting	[28,29]
	Clozapine	Treatment-resistant schizophrenia	Orthostatic hypotension, cardiomyopathy	Induces autophagy and apoptosis in breast cancer cells through ROS production	[3]
	Quetiapine	Schizophrenia, bipolar disorder	Hypotension, neuroleptic malignant syndrome (NMS)	As adjuvant agent with radio- and chemotherapy in triple-negative breast cancer (in vitro). Suppresses progression in hepatocellular carcinoma (in vivo)	[30,31]
	Lurasidone	Schizophrenia, bipolar depression	Orthostatic hypotension, tardive dyskinesia	Sensitizes lung and pancreatic cancer cells to osimertinib through induction of autophagy and reduction in survivin expression	[32,33]
	Pimavanserin	Parkinson’s psychosis	QT prolongation	Induces autophagy-mediated apoptosis in pancreatic cells and xenograft mouse model as well as triple negative breast cancer cells	[13,34]
	Lumateperone	Schizophrenia	Neuroleptic malignant syndrome (NMS), suicidal ideation	No data available on cancer	[35]
	Brexpiprazole	Schizophrenia, adjunct antidepressant	Tardive dyskinesia, sedation, weight gain	Inhibits cancer cells and depletes CSCs in glioblastoma, pancreatic cancer, and lung cancers in vitro and in a xenograft mouse model. It also inhibits migration of colorectal cancer cells	[36,37]
Atypical (3rd Generation)	Aripiprazole	Schizophrenia, bipolar disorder, irritability associated with autistic disorder	Akathisia, Extrapyramidal symptoms (EPS)	Specifically inhibits growth of cancer and CSCs, increases CSC marker expression, and enhances chemotherapeutic sensitivity	[12,38]
	Cariprazine	Schizophrenia, bipolar disorder	Cerebrovascular events, weight gain	Induces apoptosis in cervical and colon cancer cells; reduces glioblastoma growth in vitro and in vivo through D2R/D3R/ARRB2 axis modulation	[39,40,41]

**Table 2:** List of antipsychotic-associated antitumor effects in multiple malignancies.

Antipsychotic	Main Anticancer Effects	Key Mechanisms/Pathways	Cancer Types Mentioned	References
Thioridazine (TDZ)	• Selectively eliminates CSCs • Suppresses proliferation • Induces apoptosis/autophagy in multiple models	• Dopamine receptor antagonism • PI3K/Akt signaling • Bcl-2 family regulation • Autophagy-dependent survival disruption	• Glioblastoma • Breast cancer • Ovarian cancer • Osteosarcoma • Gastric cancer	[23,46,61,62,63]
Chlorpromazine (CPZ)	• Induces apoptosis and autophagy • Inhibits survival and proliferation • May sensitize tumors to therapy	• Mitochondrial membrane potential disruption • Increases ROS and suppresses PI3K/Akt/mTOR • Inhibits clathrin-mediated endocytosis • Interferes with DNA repair	• Glioma • Hepatocellular carcinoma	[10,17,64]
Pimozide (PZ)	• Suppresses proliferation • Increases apoptosis • Inhibits migration and invasion	• D2 receptor blockade • STAT5 inhibition • STAT3 inhibition • RAF/ERK1/2 modulation • Autophagy enhancement	• Prostate cancer • Breast cancer • Hematologic and solid malignancies	[11,25,26,65]
Risperidone (RD)	• Inhibits proliferation and induces apoptosis; possible effects on angiogenesis and tumor microenvironment.	• D2 receptor antagonism and downstream signaling modulation	• Breast cancer	[27]
Olanzapine (OP)	• Sensitizes CSC and non-CSC to chemotherapy (5-fluorouracil & gemcitabine)	• Reduces survivin expression	• Lung • Pancreatic • Ovarian	[66,67]
Aripiprazole (APZ)	• Suppresses proliferation • Induces apoptosis • Overcomes chemoresistance • Targets CSCs	• PI3K/Akt/mTOR; Wnt/β-catenin • Mitochondrial dysfunction • ER stress • P-glycoprotein inhibition	• Multiple preclinical cancer models • CSC-related studies	[12,46]
Brexpiprazole (BPZ)	• Suppresses tumor growth and induces apoptosis • Sensitizes stem-like cells to targeted therapy	• Downregulation of survivin • Reduced Sox2 and CSC markers • Synergy with EGFR inhibitors	• Glioblastoma • Pancreatic cancer • Lung cancer	[36]

**Table 3:** Selected Clinical Studies of Antipsychotics in Oncology.

Drug	Study Type	Key Findings	Reference
**Thioridazine**	Phase I (NCT02096289)	Reduced LSCs; QT-limited dosing	[70]
**Chlorpromazine**	Phase II (NCT04224441)	Treatment of glioblastoma patients with an unmethylated *MGMT* gene promoter using TMZ + CPZ reduced tumor progression	[71]
**Aripiprazole**	Cohort study	Reduced breast cancer incidence	[72]

RCT: randomized controlled trial; LSCs: leukemia stem cells; TMZ: temozolomide.

**Table 4:** Safety Considerations Across Antipsychotic Classes.

Drug Class	Representative Agents	Key Safety Profile	Common Adverse Effects	Serious Adverse Effects	Notes	Reference
First-Generation Antipsychotics	Chlorpromazine, Haloperidol, Thioridazine, Pimozide, Trifluoperazine	Strong D2 blockade; high EPS risk; anticholinergic and cardiovascular toxicity	Sedation, dry mouth, constipation, blurred vision, dizziness	EPS, NMS, QT prolongation, Torsades de pointes, hepatotoxicity, seizures	QT prolongation limits dose escalation; many anticancer effects require supratherapeutic dosing; careful ECG monitoring needed	[15,23,70,71]
Second-Generation Antipsychotics	Olanzapine, Risperidone, Quetiapine, Clozapine, Lurasidone, Ziprasidone	Mixed D2/5-HT2A antagonism; lower EPS risk; higher metabolic burden	Weight gain, hyperglycemia, dyslipidemia, sedation, orthostatic hypotension	Metabolic syndrome, myocarditis (clozapine), agranulocytosis (clozapine), QT prolongation (ziprasidone), NMS	Metabolic toxicity may worsen cancer-related cachexia or steroid-induced hyperglycemia; olanzapine is widely used for CINV	[29,33,88,89,90]
Third-Generation Antipsychotics	Aripiprazole, Brexpiprazole, Cariprazine, Lumateperone	D2 partial agonism; lower EPS and metabolic risk; improved tolerability	Akathisia, headache, insomnia, nausea, mild weight changes	NMS, EPS (rare), hypotension, seizures, suicidal ideation	Better safety window for repurposing; anticancer doses may still exceed psychiatric ranges; favorable for long-term adjunctive use	[91,92,93,94]

NMS = neuroleptic malignant syndrome; EPS = extrapyramidal symptoms.

**Table 5:** Antipsychotic-Guided Biomarkers: Targets, Cancer Types, and Evidence Level.

Biomarker	Type/Category	Cancer(s)/Context	Responsive Antipsychotic(s)	Predictive Relationship	Reference
D2R (Dopamine Receptor D2) overexpression	Receptor expression	Glioblastoma	Phenothiazines, Butyrophenones	Overexpression linked to D2R-dependent tumor cell effects; findings on direct correlation with drug sensitivity are mixed	[96]
D2R overexpression	Receptor expression	Breast cancer	Phenothiazines, Butyrophenones	Overexpression reported across ER/PR/HER2 subtypes, highest in triple-negative cells	[97]
D2R overexpression	Receptor expression	Leukemia (AML)	Phenothiazines (thioridazine)	D2R identified on leukemic stem cells; proposed as a cross-malignancy biomarker	[46]
Sox2 (CSC marker)	Cancer stem cell marker	Pancreatic cancer CSC xenograft model (preclinical)	Brexpiprazole	Sox2 and survivin expression reduced with tumor growth suppression	[36]
CD44/ALDH1 (CSC markers)	Cancer stem cell marker	Leukemic and breast cancer stem cells (preclinical)	Thioridazine	General CSC-selective modulation; simultaneous CD44/ALDH1 reduction not confirmed in a single dedicated source	[46]
LC3B accumulation (autophagy)	Autophagy-related signature	Glioma (U-87MG)	Chlorpromazine	LC3-II accumulation and Akt/mTOR inhibition accompany autophagic cell death and tumor suppression	[10]
LC3B accumulation/impaired lysosomal acidification	Autophagy-related signature	Glioblastoma cancer stem cells (preclinical)	Thioridazine	Autophagy induction (LC3 expression) observed in CSC models	[46]
STAT5 activation	Signaling pathway activation	Chronic myelogenous leukemia (CML)	Pimozide	Dose-dependent loss of activating STAT5 tyrosine phosphorylation predicts response	[26]
STAT3 activation (phospho-STAT3 Tyr705)	Signaling pathway activation	Triple-negative breast cancer	Pimozide	Baseline pSTAT3 (Tyr705) status predicts sensitivity to invasion/migration inhibition	[95]
STAT3 activation (phospho-STAT3 Tyr705)	Signaling pathway activation	Glioblastoma and medulloblastoma (brain tumors)	Pimozide	pSTAT3 (Tyr705) suppression accompanies autophagy induction and tumor growth inhibition	[98]

## Data Availability

The datasets generated and/or analyzed during the current study are available upon request.

## Abbreviations

The following abbreviations are used in this manuscript:

5-HT2A	5-hydroxytryptamine receptor 2A
Akt	Protein kinase B
AI	Artificial Intelligence
AML	Acute myeloid leukemia
APZ	Aripiprazole
BPZ	Brexpiprazole
CINV	Chemotherapy-induced nausea and vomiting
CPZ	Chlorpromazine
CSC	Cancer stem cell
D2R	Dopamine D2 receptor
EGFR	Epidermal growth factor receptor
EPS	Extrapyramidal symptoms
ERK	Extracellular signal-regulated kinase
G-CSF	Granulocyte colony stimulating factor
GPX4	Glutathione Peroxidase 4
FDA	U.S. Food and Drug Administration
LSCs	Leukemia stem cells
MAPK	Mitogen-activated protein kinase
mTOR	Mammalian target of rapamycin
NMS	Neuroleptic malignant syndrome
Nrf2	Nuclear factor erythroid 2-related factor 2
OP	Olanzapine
PI3K	Phoshoinositide-3-kinase
PZ	Pimozide
RCT	Randomized controlled trial
RD	Risperidone
ROS	Reactive oxygen species
SLC7A11	Solute carrier family 7 member 11
Sox2	Sex determining region Y-box 2
TD	Tardive dyskinesia
TDZ	Thioridazine
VEGF	Vascular endothelial growth factor
Wnt	Wingless and Int-1
YAP	Yes-associated protein
